# Changing the diagnostic paradigm for sugarcane: development of a mill-based diagnostic for ratoon stunting disease in crude cane juice

**DOI:** 10.3389/fpls.2023.1257894

**Published:** 2023-10-12

**Authors:** Sriti Burman, Michael G. Mason, Jessica Hintzsche, Yiping Zou, Lucy Gibbs, Laura MacGillycuddy, Robert C. Magarey, José R. Botella

**Affiliations:** ^1^ Plant Genetic Engineering Laboratory, School of Agriculture and Food Sustainability, The University of Queensland, Brisbane, QLD, Australia; ^2^ Sugar Research Australia, Brisbane, QLD, Australia; ^3^ Sugar Research Australia, Tully, QLD, Australia

**Keywords:** ratoon stunting disease, RSD, sugarcane, crop diagnostics, LAMP, DNA dipsticks

## Abstract

The availability of efficient diagnostic methods is crucial to monitor the incidence of crop diseases and implement effective management strategies. One of the most important elements in diagnostics, especially in large acreage crops, is the sampling strategy as hundreds of thousands of individual plants can grow in a single farm, making it difficult to assess disease incidence in field surveys. This problem is compounded when there are no external disease symptoms, as in the case for the ratoon stunting disease (RSD) in sugarcane. We have developed an alternative approach of disease surveillance by using the crude cane juice expressed at the sugar factory (mill). For this purpose, we optimized DNA extraction and amplification conditions for the bacterium *Leifsonia xyli* subsp *xyli*, the causal agent of RSD. The use of nucleic acid dipsticks and LAMP isothermal amplification allows to perform the assays at the mills, even in the absence of molecular biology laboratories. Our method has been validated using the qPCR industry standard and shows higher sensitivity. This approach circumvents sampling limitations, providing RSD incidence evaluation on commercial crops and facilitating disease mapping across growing regions. There is also potential is to extend the technology to other sugarcane diseases as well as other processed crops.

## 1 Introduction

Sugarcane is an important commercial crop accounting for 20 percent of the global agricultural crop production (1.9 billion tonnes) and provides multiple end products, including raw and white sugar, ethanol, molasses and bagasse (OECD et al., 2021). Plant pathogens and pests pose very significant biosecurity risks to the sugarcane industry; with factors like passenger and freight movements, and climate change accelerating the spread of previously endemic or unknown diseases. Ratoon Stunting Disease (RSD) is a ubiquitous and highly contagious bacterial disease of sugarcane, caused by a xylem limited bacterium *Leifsonia xyli* subsp *xyli* (Lxx here after) (Gillaspie et al., 1973; Davis et al., 1980; Gillaspie and Teakle, 1989). Sugarcane is vegetatively propagated from cuttings, with 12-month plant crops followed by annual harvests of ‘ratoon crops’ from the same planting (Hoy et al., 1999). Diseased plant crops inevitably lead to diseased ratoon crops, making disease-free plant establishment even more important. Lxx disseminates either by planting infected ‘seed’ cane or via mechanical means e.g. by implements contaminated by infected juice during harvests (Damann and Benda, 1983; Davis and Bailey, 2000; Comstock, 2002). RSD does not present distinguishable external symptoms, especially when bacterial titers are low, often resembling abiotic stress symptoms like the stunting observed during drought seasons. The effects of RSD on crop yield are also more pronounced in dry seasons and in ratoon crops (Davis and Bailey, 2000). For logistical reasons, disease detection largely focuses on plant nurseries which provide vegetative planting material to establish new crops (Young et al., 2016). Disease-free plant sources help to ensure that crops are established RSD-free, a critical management strategy. Very little information is available on RSD incidence in commercial crops, given the logistical limitations associated with the sampling needed to conduct whole farm surveys. In some Australian sugarcane cropping regions, disease incidence may exceed 30% of crops with associated yield losses ranging between 5-60%; with many farmers unaware of the presence of RSD (Magarey et al., 2021; SRA, 2021). The core management strategy to control the disease includes disease-free seed cane, equipment sterilization and a fallow period devoid of diseased sugarcane plants. The correct and timely detection of the disease provides for assessment of the success or failure of disease management, a critical issue.

Available methods for RSD diagnosis include direct detection of Lxx by phase contrast microscopy and bacterial cultivation; or indirect detection by serological assays like dot blot immunoassay (DBI), evaporative binding enzyme assay (EB-EIA), immunofluorescence microscopy (IFM) and nucleic acid amplification (NAA) based methods like polymerase chain reaction (PCR), quantitative PCR (qPCR), nested PCR and loop mediated isothermal amplification (LAMP), with NAA based methods being the most sensitive (Teakle et al., 1973; Davis et al., 1980; Croft et al., 1994; Croft et al., 2004; Grisham et al., 2007; Liu et al., 2013; Ghai et al., 2014). Importantly, all the above mentioned methods rely on plant extracts collected from individual sugarcane stalks, severely constraining disease detection in commercial crops (Hoy et al., 1999; Grisham et al., 2007; Ghai et al., 2014; Young et al., 2016) The effectiveness of any RSD diagnostic technology for commercial crops hinges not only on the sensitivity of the assay, but also adequate sampling and accurate representation of the disease prevalence across a farm which typically >80,000 sugarcane stalks per hectare.

Almost every sugar factory around the world crushes cane, extracts the stalk juice, and assays for juice quality. In the Australian industry, every batch (rake) of harvested cane delivered to the sugar factory has associated geographic information system data allowing to relate yield, sugar content, moisture and fiber to be related to individual farm blocks, sub-districts, varieties, planting and harvester contractor groups, soil types, crop cycle information and management practices. Therefore, a diagnostic assay able to detect Lxx in the juice extracted at the mill would overcome most of the limitations of the current RSD incidence detection strategies. Using this sampling strategy, RSD prevalence could be accurately determined across a farm, as opposed to current methods which are largely restricted to planting material with only 16-20 stalks per farm (Young et al., 2016).

With the xylem sap or leaf sheath biopsies being the principal plant extract assayed in the routine qPCR detection systems currently utilized within industry, the presence of amplification inhibitors does not present an issue. The focus of the research presented here is the RSD detection in juice extracted from whole stalks in a rake, after crushing between rollers at the sugar factory. The most challenging issue for developing a NAA based assay using juice extracted from whole stalks after crushing at the sugar factory is the presence of high sugar concentrations, soil debris, humic acid and other contaminants that inhibit efficient DNA amplification. In this work, we have developed a diagnostic method combining fast and efficient DNA purification with isothermal LAMP for amplification of Lxx genomic DNA. A cellulose based nucleic acid purification method, previously developed by our group allows the binding of nucleic acids from complex biological samples (Zou et al., 2017; Mason and Botella, 2020; Mason et al., 2020). This method for DNA purification requires 30 seconds for sample processing, without the need for pipettes or specialized laboratory equipment. Amongst the available NAA based diagnostic techniques, LAMP is one of the simplest, due to its isothermal nature and relative tolerance to the presence of inhibitors, making it well suited for point-of-care (POC) diagnostics in agriculture and medicine (Notomi et al., 2000; Notomi et al., 2015; Lau and Botella, 2017; Panno et al., 2020; Zou et al., 2020; Zhu et al., 2022). The use of LAMP for Lxx detection has been reported in the literature with sensitivity comparable to other conventional methods like qPCR (Liu et al., 2013; Ghai et al., 2014; Wu et al., 2018).

No previous research has been undertaken with respect to detection of *Leifsonia xyli* subsp. xyli in rakes of cane producing first expressed sugarcane juice, especially with the intent to develop the diagnostic technology for POC needs at a sugar mill, which was the main focus of this study.

## 2 Materials and methods

### 2.1 Primer design

Blast searches of the annotated genes of Lxx against publicly available microbial genomes revealed that the gene coding for the DNA replication/repair protein RecF (Accession: AE016822; locus tag: LXX_RS00020) was unique and conserved across Lxx genomes and thus used as a potential target for Lxx detection. 5 LAMP primer sets, named LXX20-1, LXX 20-2, LXX20-3, LXX20-4 and LXX20-5 were subsequently designed. Previously published gene encoding the UDP-N-acetylglucosamine 2-epimerase (Accession: AE016822; locus tag: LXX14026) was also used as a target for LAMP primer development (Taylor et al., 2003). LAMP primers were designed using the LAMP primer development software Primer Explorer (https://primerexplorer.jp/e/) and following the guidelines by Notomi et al. (Notomi et al., 2000). 16S rRNA primers previously reported by Liu et al. were also used in this study (Liu et al., 2013). The LAMP primers were also checked for self-priming using Thermo Fisher Scientific’s primer analyzer tool(https://www.thermofisher.com/au/en/home/brands/thermo-scientific/molecular-biology/molecular-biology-learning-center/molecular-biology-resource-library/thermo-scientific-web-tools/multiple-primer-analyzer.html). Details of all six primer sets are available in **Table 1**.

**Table 1:** List of Lxx LAMP primers used in this study.

Primer	Primer Sequence (5’-3’)
Lxx20-1F3	GCACCCGTTCGTATTCGG
Lxx20-1B3	AGCTGAACCGCGGAGCG
Lxx20-1FIP	TGTCCGCCGGCGCTTTCT-GATCACCGCTGACAAACGG
Lxx20-1BIP	CCACGGACGAGGGCCAGAT-CGTGCTCAGGTCAATCGG
Lxx20-2F3	GGGTTCCCCACGGACGAG
Lxx20-2B3	GCTCTCGGATCGCATCGG
Lxx20-2FIP	TGCTCAGGTCAATCGGGCC-ACACGCTCGAAAAGTACCGC
Lxx20-2BIP	CCTCGACCAGAAGCTCCCGCGCT-TCGAGCGACCAAGCCA
Lxx20-3F3	CGCGAACAACACGCTCGA
Lxx20-3B3	CAATGGCCAGGGAAAGACC
Lxx20-3FIP	GCAGCTGAACCGCGGAGCGGC-CTTGATCGCGGCCCGA
Lxx20-3BIP	GCCCGGACGACAGCCAACT-GGTTACCTGAGCGCTCTCG
Lxx20-4F3	TCCCGGAAACGGGACG
Lxx20-4B3	GGCCGCGATCAAGCC
Lxx20-4FIP	TCCCCGTTTGTCAGCGGTGAT-CAGAGTGTTCCGCTGTTTCAG
Lxx20-4BIP	CGCTGGATGAGGAGCTGGTC-GGTACTTTTCGAGCGTGTTGTTC
Lxx20-5F3	GCGATCACCGCTGACAA
Lxx20-5B3	CGCGGGAGCTTCTGGT
Lxx20-5FIP	TCCGTGGGGAACCCGGTGT-GGGGATTCCGCTGGATGAG
Lxx20-5BIP	CAGATCCTCCGGCGCGAAC-GGAGCGGCCAATCGTG
S1000-2F3	CCATAGCGTTCCTGGGAGA
S1000-2B3	CCGACCCGAGATTATCAAGC
S1000-2FIP	CGGGATCGGGGCACCTAACT-ATCTGTCTCGTGCGATTCTCA
S1000-2BIP	CTTCAAAGAAGGACCCCGCCA-GTTGGGAAACCGGGCAAT
S1000-2LF	TTATCCTCGAAGGCGTTGGCG
S1000-2LB	TCGTAGTGCTGTCCAGAATGGATC

### 2.2 Sample collection and DNA purification

15mL crude juice samples were collected at two sugar factories and processed immediately or stored at -20°C until processed later. Lxx DNA was purified from these samples using the dipstick DNA technique as described by Zou et al. and Mason et al. (Zou et al., 2017; Mason and Botella, 2020). Briefly, the exposed cellulose DNA binding zone of the dipstick was dipped in the crude juice, following which it was soaked for 2 minutes in 500μL extraction buffer (20mM Tris (pH 8), 25mM NaCl, 2.5mM EDTA, 0.5% Sodium Dodecyl Sulfate (w/v), 2% PVP (w/v)); dipped 10x times in 800μL wash buffer (10mM Tris (pH 8.8), and 8mM MgCl_2_) and any bound DNA was eluted directly into the reaction tube containing 45μL LAMP reagents.

For total DNA extraction, frozen crude juice samples were thawed and allowed to sediment at room temperature for 30 minutes, followed by heat lysis of equal volumes of the sample in the presence of lysis buffer (4% SDS (w/v), 500mM NaCl, and 50 mM EDTA) at 65°C for 30 minutes. The total DNA was then purified using SPRI beads (AMPure XP, Beckman Coulter, Australia) according to manufacturer’s instructions and stored at -20°C until further use.

### 2.3 Lxx LAMP amplification

Unless stated, LAMP reaction mixture contained 0.8M betaine, 20mM Tris-HCl (pH 8.8), 50mM KCl, 10mM (NH_4_)_2_SO_4_, 0.1% Tween^®^ 20, 8mM MgSO_4_, 1.2mM dNTP (each), 0.32 U/mL Bst 2.0 warm start polymerase (New England Biolab), 0.2μM (each) F3 and B3 primers, 0.8μM (each) loop forward and reverse primer, 45 μL of reactions were incubated at 65°C for 60 minutes in our custom made hand held diagnostic device (Botella, 2022). When necessary, the amplified products were visualized using agarose gel electrophoresis, the raw values and real time curves were analyzed using Microsoft Excel. In case of fluorescent LAMP reactions, Syto9 dye was added at a final concentration of 1.25 μM and reactions incubated at 65°C for 60 minutes in a Bio-Rad CFX 96™ Real-Time System (Bio-Rad Laboratories, Australia).

### 2.4 Lxx qPCR and PCR amplification

The real-time quantitative PCR (qPCR) reactions were performed in a Bio-Rad CFX 96™ Real-Time System (Bio-Rad Laboratories, Australia) and were performed in 96-well plate, with three technical replicates for each sample. Each 25 μL reaction mixture contained 12.5 μL of GoTaq® qPCR master mix (2X) (Promega, Australia), 1 μM each of Lxx202FB/Lxx331R, 2 μL of total DNA template (extracted as described above), 5.5 μL ultra-pure water. The standard curves were generated using a serial dilution of the 130-bp purified amplicon of the Lxx 16S-23S rRNA intergenic transcribed spacer (ITS) region as described by Grisham et al.(Grisham et al., 2007). The no template control contained 2 μL of ultra-pure water. Thermal cycling consisted of denaturation at 95°C for 5 minutes, followed by 40 cycles of 95°C for 10 seconds, 60°C for 30 seconds and 72°C for 20 seconds. Post amplification final melting curve of 65°C to 97°C was also performed. The efficiency of primers was calculated according to Rasmussen et al.(Rasmussen, 2001). PCR amplification of the Lxx UDP-N-acetylglucosamine 2-epimerase and Lxx 16S-23S rRNA ITS gene was performed in 20 μL reaction volumes, with 10 μL GoTaq® green master mix (2X) (Promega, Australia), 0.5 μM each of S1000-2 F3/B3 primers and 2 μL of total DNA from crude juice or xylem extracted from infected plant. Cycling conditions were as following: 95°C for 2 minutes; 35 cycles of 95°C for 30 seconds, 60°C for 30 seconds, 72°C for 30 seconds; and a final extension at 72°C for 5 minutes. The PCR products were visualized by agarose gel electrophoresis.

### 2.5 Comparison of LAMP and qPCR Lxx sensitivity

The 230-bp amplicon of UDP-N-acetylglucosamine 2-epimerase gene (for LAMP reaction) and 130-bp amplicon of Lxx 16S-23S rRNA ITS gene (for qPCR) were purified using QIAquick PCR kit as per manufacturer’s instruction (Qiagen, Australia). For LAMP, 10^6^, 10^5^, 10^4^, 10^3^, 10^2^, 10 and 1 fg of purified DNA was added to clean sugarcane juice sample, DNA was purified using the dipstick method as described in section 2.2 and eluted into LAMP reaction of 45 μL each. For qPCR, 4x10^4^, 4x10^3^, 4x10^2^, 40 and 4 fg of DNA was directly added to each qPCR reaction tube of 25 μL volume, with three technical replicates for each DNA concentration. These concentrations of Lxx DNA were also used for generation of standard curves for qPCR. The thermal conditions and composition of the amplification reactions were as described in Sections 2.3 and 2.4.

### 2.6 Statistical analyses

GraphPad Prism (version 9.5.1) was used for all statistical analyses. For calculation of LAMP and qPCR diagnostic sensitivity, specificity, and area under the curve (AUC), the ROC curve analysis embedded in the software was used. When comparing the outcomes of two or methods, a chi-square test with 95% confidence interval was applied. One-way ANOVA followed by Dunn’s multiple comparison test was applied when comparing differences between different LAMP reaction composition parameters. Graphical rendering of data and schematics was done using Microsoft Office suite, GraphPad Prism and BioRender (BioRender.com).

## 3 Results

### 3.1 Lxx-specific primer design and confirmation

**Figure 1:**
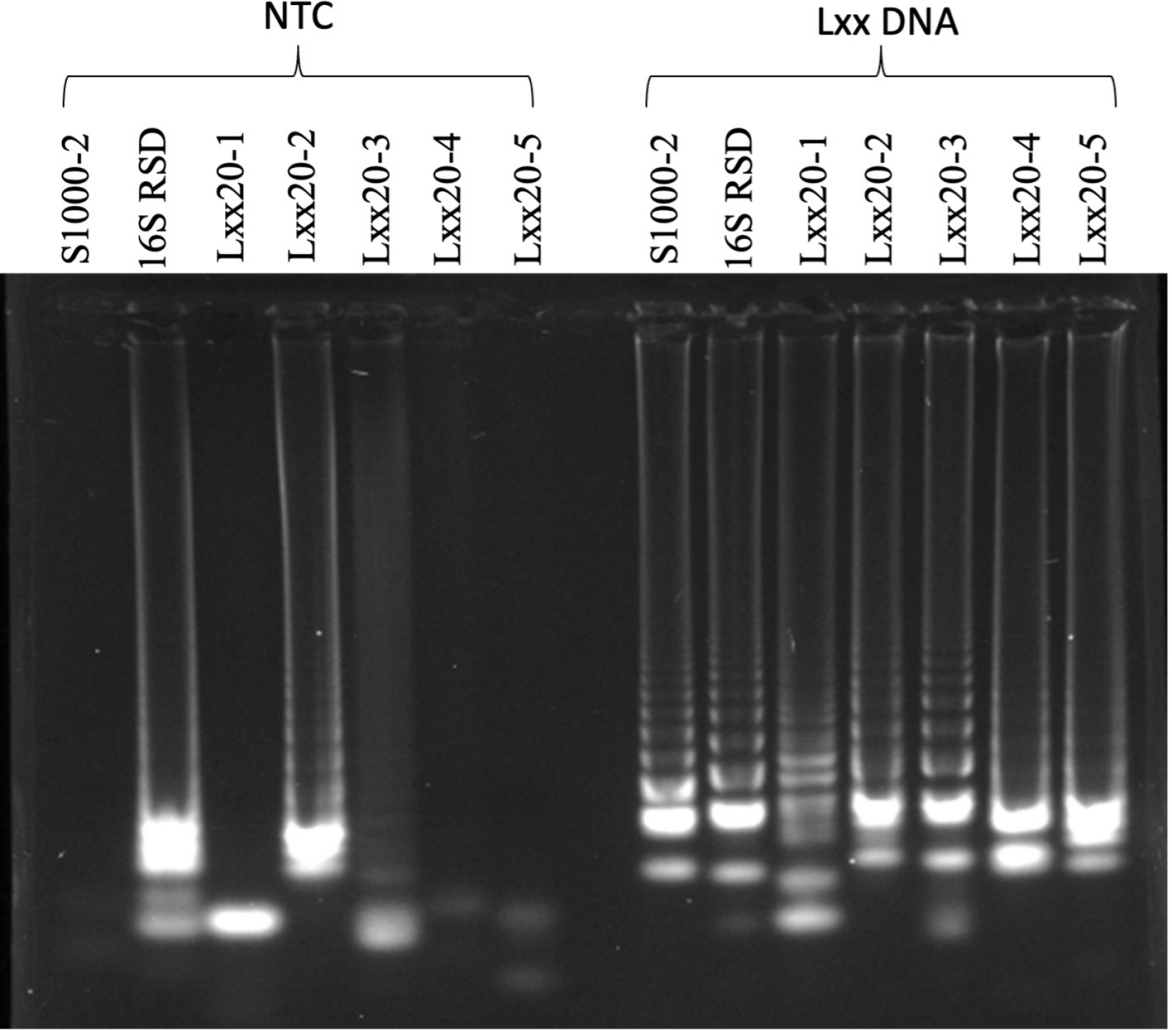
Characterization of LAMP primers: Seven different LAMP primer sets were used for amplification using purified Lxx DNA or water (NTC). LAMP reactions were incubated at 65°C for 70 minutes and 6 μL of reaction loaded in an electrophoresis gel.

Five LAMP primer sets, named LXX20-1, LXX20-2, LXX20-3, LXX20-4 and LXX20-5 were designed targeting the DNA replication/repair protein RecF (Accession: AE016822; locus tag: LXX_RS00020) (**Table 1**). In addition, a LAMP primer set named S1000-2 was designed targeting a gene encoding the UDP-N-acetylglucosamine 2-epimerase (Accession: AE016822; locus tag: LXX14026)(Taylor et al., 2003). Finally, a primer set (called 16S RSD) published by Liu et al. was also evaluated (Liu et al., 2013). All seven LAMP primer sets were tested for their ability to yield amplification products using purified Lxx DNA and their susceptibility to produce amplification products by self-priming in the absence of Lxx DNA (i.e. no template controls or NTCs). In the absence of Lxx DNA (**Figure 1**, left side), primer sets 16S RSD, LXX20-1, LXX20-2, LXX20-3, LXX20-4 and LXX20-5 generated amplification bands of different intensities; whereas primer set S1000-2 failed to produce any detectable amplification. All 7 primer sets produced strong amplification in the presence of Lxx DNA (**Figure 1**, right side), evidenced by the characteristic LAMP ladder-like bands. Based on these results, the primer set S1000-2 was selected for further experiments.

The S1000-2 primers F3/B3 were used in PCR reactions and the sequence identity of the PCR amplicons confirmed using Sanger sequencing. The ability of the S1000-2 F3/B3 primers to detect different *L. xyli* subsp xyli strains was established by testing xylem extracts from plants from suspected RSD positive farms from 13 different growing locations across the Australian sugarcane industry (**Supplementary Table 1**). The S1000-2F3/B3 primers successfully amplified a 230bp amplicon in 11 out of the 13 samples analyzed (**Supplementary Figure 1**), and Sanger sequencing revealed strong sequence conservation among all isolates. The specificity of the S1000-2 primers towards Lxx UDP-N-acetylglucosamine 2-epimerase gene was ascertained by sequencing a 230bp amplicon in total DNA extracted from a Lxx infected crude juice sample (**Supplementary Figure 2**). The sequencing results revealed high percentage of sequence identity to Lxx strain CTCB07 genome and low sequence homology to other prevalent environmental bacteria.

### 3.2 Initial optimization of sample processing in mill-generated sugarcane crude juice

**Figure 2:**
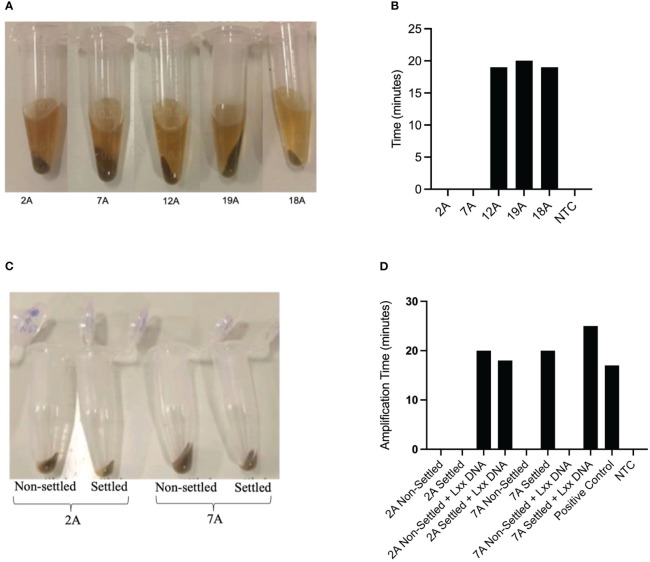
Initial sampling optimization. **(A)** Pellet sizes of five 500µL sugarcane mill juice samples centrifuged at 16,000 × g for 5 minutes, **(B)** Samples shown in **(A)** were resuspended in 500µL of extraction buffer and used for Lxx DNA amplification by LAMP. The graph shows time to amplification. **(C)** Samples 2A and 7A, which did not produce amplicons using the centrifugation method, were allowed to settle for 60 minutes and the supernatant transferred to a fresh tube prior to centrifugation, reducing the quantity of silt in the samples; **(D)** LAMP amplification of Lxx in samples with and without the pre-settling step. The same samples were also analyzed after spiking the original Mill crude juice with xylem from an infected plant.

Sugar mill laboratory staff has limited time for sample processing and are not usually proficient in molecular biology techniques. We therefore decided to use the dipstick DNA purification method as it combines simplicity with speed (Zou et al., 2017; Mason and Botella, 2020). Crude mill juice samples contain diverse plant and non-plant materials, high concentrations of sugar, silt and soil which exert a strong inhibitory effect on DNA amplification (Henson and French, 1993; Lopes and Damann, 1993). Crude mill juice samples can also show large variability in the quantity of silt/soil present as can be seen in the different amounts of pellet observed after centrifugation of five independent samples, obtained from a region with high incidence of RSD (**Figure 2A**). In an initial approach, 500µL of crude juice samples were centrifuged for 5 mins at 16,000 x g to collect bacterial cells before discarding the supernatant and resuspending the pellet in the same volume of extraction buffer. DNA dipsticks were used to purify DNA from the solution and release it into LAMP reactions containing the S1000-2 primer set for Lxx detection. Using this method, three out of five samples analyzed produced positive LAMP amplification products 18-20 minutes after starting the reaction (**Figure 2B**). The negative amplification results obtained for samples 2A and 7A could be due either to the absence of Lxx bacteria in the juice or the presence of inhibitors in the amplification reaction. In an effort to reduce the amount of insoluble matter, 1.6 ml of crude mill juice samples 2A and 7A were allowed to settle for 60 mins before carefully collecting 500µL of sample from the top and subjecting it to the above-described treatment (i.e. centrifugation plus resuspension in extraction buffer) (**Figure 2C**). Dipstick purification of DNA from resuspended samples followed by LAMP amplification failed to detect the presence of Lxx in sample 2A, while the addition of the settlement step resulted in a positive amplification for sample 7A. Simultaneous treatments were also performed spiking the initial juice with xylem from an RSD-positive plant. In the case of sample 7A, spiking failed to produce an amplification product in the non-settled sample while settling produced a positive amplification indicating that the pre-settlement step is efficient in reducing inhibitors from the crude mill juice and improves the reproducibility of the LAMP assay. In contrast, the presence of spiked Lxx DNA was detected in the settled and non-settled treatments for sample 2A (**Figure 2D**) suggesting that the initial negative result (**Figure 2B**) was due to the absence of detectable Lxx DNA in the sample.

### 3.3 Optimization of sampling method and preliminary deployment in sugar mills

**Figure 3:**
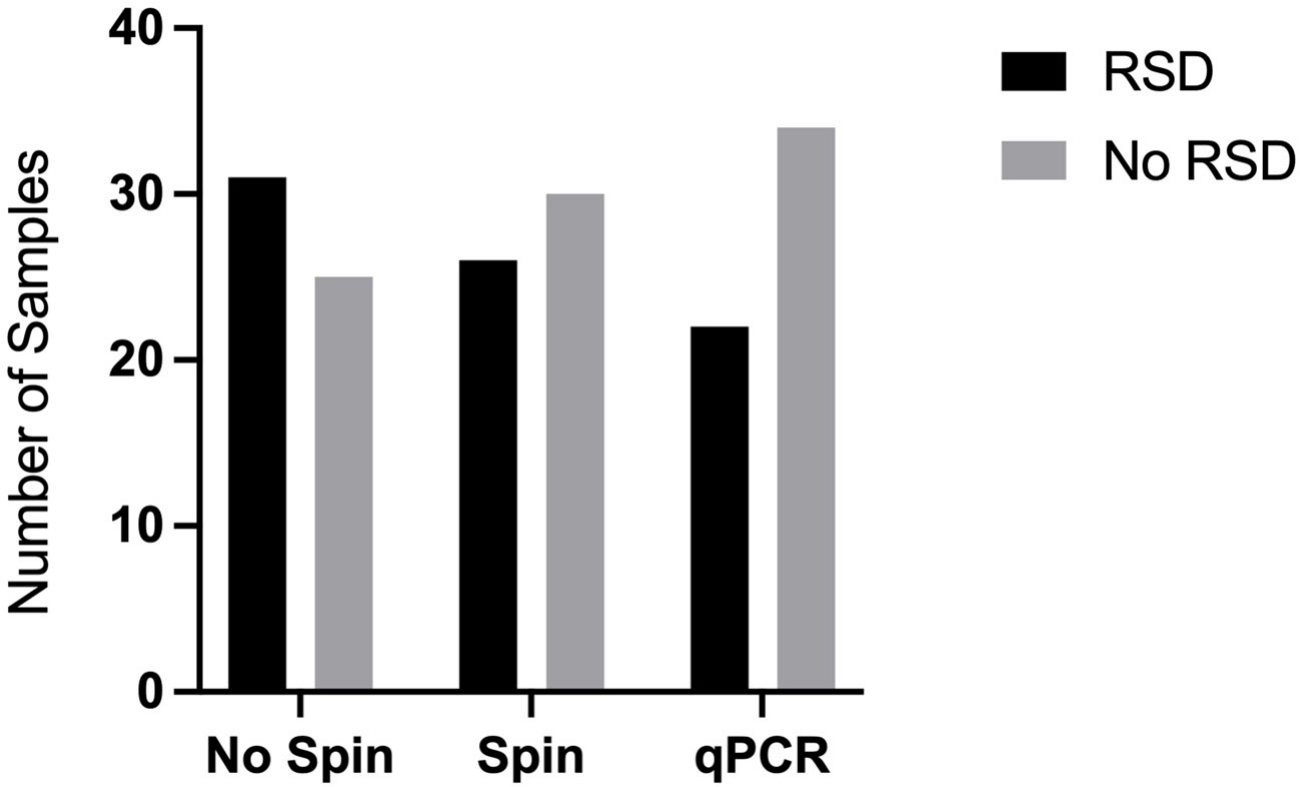
Effect of centrifugation on Lxx detection by LAMP. Mill crude juices were subjected to an initial settlement step of 60 mins followed by (a) centrifugation of samples plus resuspension (Spin) or (b) direct assay of the supernatant (No-spin). The RSD status of the samples was confirmed by extraction of the Lxx DNA using chemical and heat lysis followed by qPCR. The samples labelled RSD produced Lxx DNA amplification, whereas the samples that did not produce any Lxx amplification were designated as ‘No RSD’.

Consistent with the practical needs of a busy sugar mill with a high turnover rate of juice samples, we further investigated how to streamline the crude juice sampling process. For this purpose, we investigated whether it was necessary to centrifuge samples to collect bacterial cells or whether there was enough free Lxx DNA present in the crude juice to allow detection by LAMP amplification. We obtained 31 juice samples from a sugar mill (location undisclosed) during the 2020 crushing season and assayed them in our laboratory (*ex-situ*) using an initial settlement step of 60 mins followed by (a) centrifugation of samples plus resuspension (Spin) or (b) direct assay of the supernatant (No-spin). The RSD status of the samples was confirmed by purification of the Lxx DNA using dipsticks followed by conventional qPCR using primers previously developed for Lxx detection (Grisham et al., 2007). Both methods produced comparable results (results not shown), thus a preliminary trial at a mill was implemented during the 2021 crushing season to initiate technology transfer and to test the practical limitations of performing molecular analysis in a mill laboratory not designed for molecular biology procedures. Of the 56 juice samples assayed for RSD, 26 samples tested positive using the Spin method, whereas 31 samples tested positive using the No-Spin method, with no significant differences observed between the two methods, this experiment was repeated twice (n=2) (**Figure 3**). As the two sampling methods did not significantly affect the RSD-LAMP results, we decided to adopt the gravitational sedimentation step for 30-60 minutes followed by dipstick purification. The LAMP reaction results were validated using the qPCR method with primers described by Grisham et al., and the template for qPCR was generated by using the total DNA extraction method as described in section 2.2.

### 3.4 Optimization of LAMP reaction parameters

**Figure 4:**
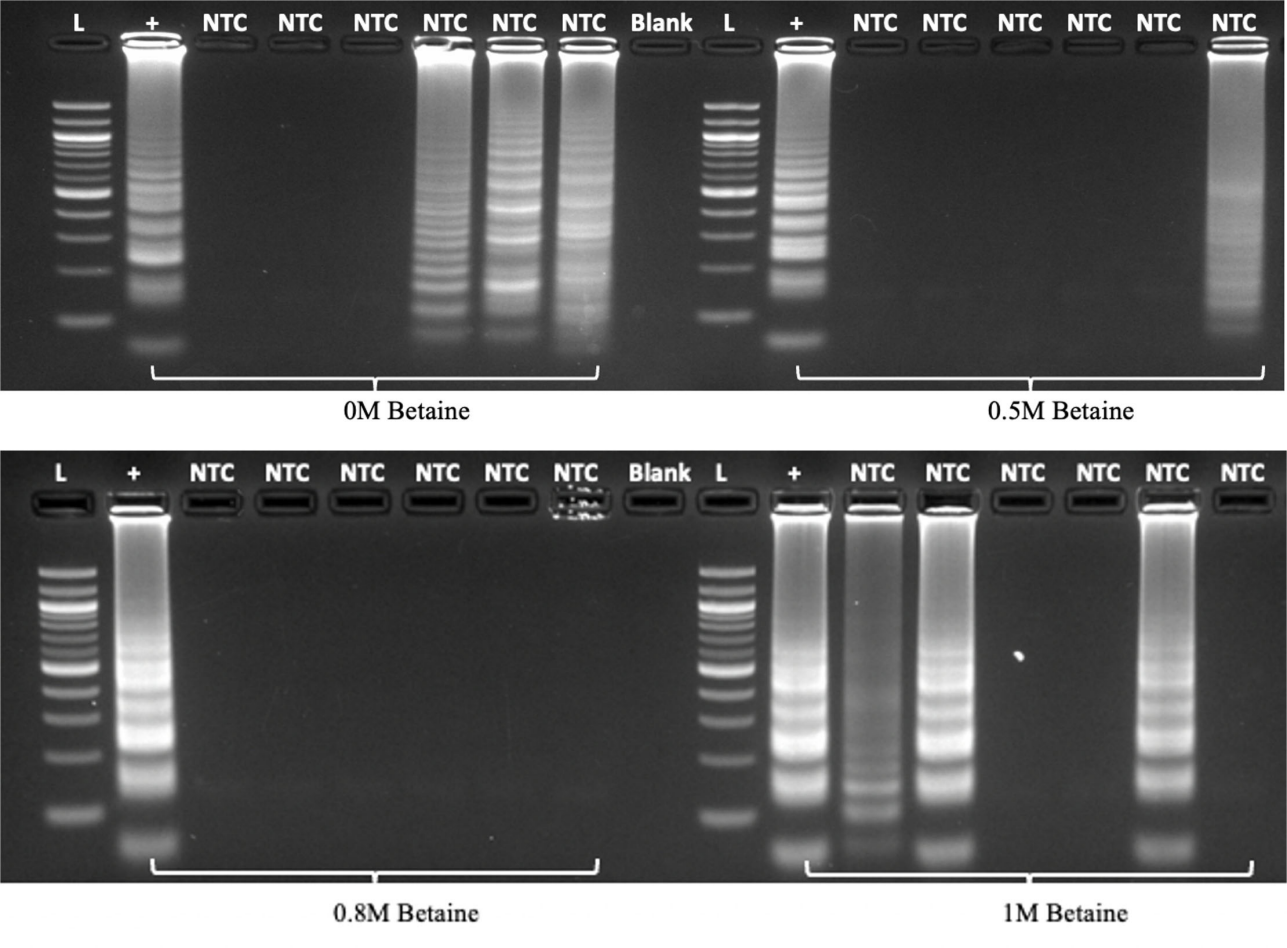
Effect of betaine concentration on LAMP specificity in the absence of target DNA (n=6). LAMP reactions were assembled using different amounts of betaine and incubated at 60°C for 60 minutes before analysis by electrophoresis. L denotes 100bp ladder; “+” denotes positive control using purified Lxx DNA; NTC denotes no template control; Blank denotes an empty lane.

To improve the robustness of the LAMP assay for Lxx detection and minimize the occurrence of non-specific false positive amplifications, we investigated the effect of betaine and primer concentrations. Previous work by our group and others have established that reducing betaine concentration in the reaction mix can significantly decrease the amplification time of the target DNA (Zou et al., 2020), hence a concentration of 0.5M betaine was used in the initial Lxx LAMP assays. One of the drawbacks of isothermal LAMP amplification is its propensity to produce non-specific amplification products due to primer dimerization in the absence of DNA template (Botella, 2022). Our experiments revealed that the use of 0.5M betaine occasionally produced self-amplification in no-template controls (NTCs) resulting in the production of false positives (**Supplementary Figure 3**), therefore we performed a betaine concentration titration experiment. Addition of 0.8M betaine to the LAMP reaction produced no amplification in water control samples (n=6), whereas 0.5M betaine produced amplification in 1 out of 6 NTCs (**Figure 4**). Excessive betaine (1M) as well as a lack of it resulted in unspecific amplification in 50% of the samples assayed.

**Figure 5:**
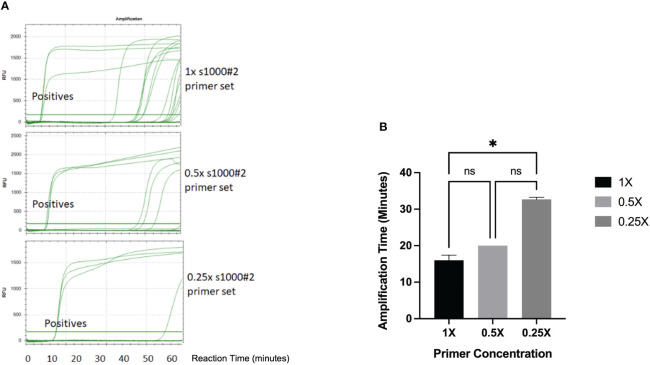
Effect of primer concentration on LAMP amplification. **(A)** 24 LAMP reactions samples containing 3 positive controls and 21 NTCs were analyzed by LAMP amplification in a real-time thermocycler using SYTO9 fluorescent dye and the standard primer concentration (upper panel), 0.5x primer concentration (middle panel) and 0.25x primer concentration (lower panel). The amplification curves for the 3 positive samples is indicated in each chromatogram. (RFU, Relative Fluorescence Units) **(B)** The turbidity of LAMP reactions containing purified Lxx DNA and different concentrations of primers was measured using portable LAMP diagnostic device. Data (n=3) was analyzed using One-way ANOVA with a *post-hoc* Dunn’s multiple comparison of means test (p<0.05). Error bars represent standard deviation. * denotes p< 0.05 and ns denotes not significant.

We also investigated the effect of primer concentration on the specificity of the LAMP amplification detected by fluorescence and turbidity-based methods. The S1000-2 primer mix was tested at the concentrations recommended by the manufacturer of the Bst 2.0 warm start polymerase used for amplification (New England Biolabs) (1.6μM FIP/BIP, 0.2μMF3/B3, 0.8μM LoopF/LoopB) (named 1x) as well as 0.5x and 0.25x. Fluorescence monitoring of the reaction in a real time thermocycler revealed that when using 1x primer concentration unspecific amplification in NTCs was first observed at a reaction time of 36.6 mins and became increasingly frequent after 50 mins (**Figure 5A**). Reducing primer concentration to 0.5x produced 3 amplifications out of 21 NTCs at incubation times of 46 minutes and above, while the use of 0.25x primer concentration produced only one false positive at ~ 60 minutes. Reduced primer concentration also conveyed a small delay in amplification. Turbidity based detection of LAMP amplification is less sensitive than fluorescence and, in our experiments, resulted in a significant delay in the detection of amplification products when using 0.25X primer concentration (p=0.0177) (**Figure 5B**). Thus, our results indicate that the likelihood of false positive amplification in the RSD assay can be significantly decreased by using a 2-fold reduction of primer concentration over that recommended by the manufacturer.

### 3.5 Validation of the RSD-LAMP assay diagnostic accuracy

**Figure 6:**
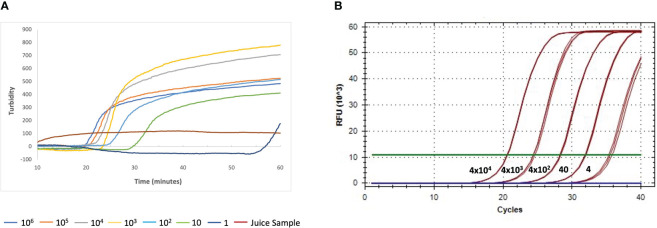
Comparison of detection sensitivity of Lxx DNA by LAMP **(A)** and qPCR **(B)**. Samples were spiked with purified PCR amplicons for the Lxx UDP-N-acetylglucosamine 2-epimerase (10^6^, 10^5^, 10^4^, 10^3^, 10^2^, 10, 1 fg) and 16S-23S rRNA ITS genes (4x10^4^, 4x10^3^, 4x10^2^, 40, 4 fg) as DNA targets for LAMP and qPCR; respectively.

We used the industry-wide accepted fluorescence-based qPCR Lxx detection method adapted from Grisham et al. (Grisham et al., 2007) to validate our RSD-LAMP assay which utilizes the dipstick method for DNA isolation and turbidity-based detection. RSD-LAMP assays were performed in samples containing serial dilutions of a fragment of the *L. xyli* subsp. *xyli* UDP-N-acetylglucosamine 2-epimerase gene (produced by PCR). LAMP produced turbidity detectable Lxx amplification with as little as 1fg of DNA within 55 minutes (**Figure 6A**), with Lxx DNA concentrations >1 fg showing amplification between 19-30 minutes. In comparison, qPCR showed reliable fluorescence-detectable amplification of 4fg of Lxx 16S-23S rRNA ITS (**Figure 6B**). These limits established for both LAMP and qPCR assays were applied as threshold cut-off for statistical analyses and evaluation of both diagnostic methods.

**Figure 7:**
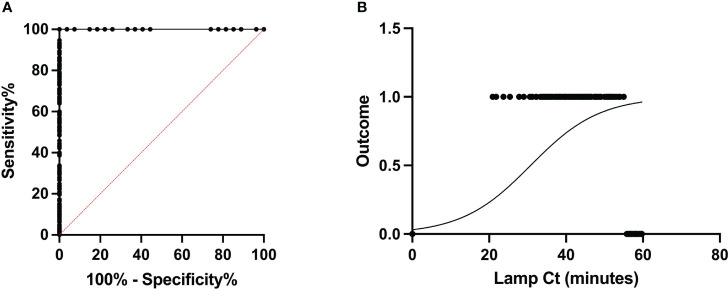
Receiver Operating Characteristic analysis of the RSD-LAMP diagnostic assay. **(A)** The ROC was computed for a total of 619 samples under non-parametric assumption, generating an AUC of 1.0. The sensitivity and specificity of the LAMP assay were 94.67% and 100% when a threshold cut-off of 55 minutes was applied, increasing the reaction time resulted in loss of specificity. **(B)** represents the logistic plot of the RSD-LAMP assay derived from odds ratio calculated from the frequency of RSD infection detected at a given LAMP Ct. Here β1 = 1.123, i.e. increasing the LAMP reaction time by 1 minute beyond 55 minutes incubation would only increase the chance of a sample being RSD positive by 1.123 times.

During the 2021 crushing season, a large-scale experiment was performed on-location at two different mills. A total of 619 juice samples from individual rakes were assayed for RSD (518 at Site A and 101 at Site B), using the optimized sampling and reaction conditions. In short, crude juice samples were left to settle for 45 minutes before using DNA dipsticks to purify the DNA and load LAMP reactions. Amplification was monitored by the appearance of turbidity using a purpose-made light reading device (Botella, 2022). Based on the limit of detection of 55 minutes for LAMP, samples showing amplification within 55 minutes in LAMP reactions were deemed as infected, whereas samples showing no amplification or late amplification (after 55 minutes) were classified as healthy. After LAMP analysis at the mills, samples were brought back to the laboratory, DNA was purified using heat and chemical lysis, followed by magnetic bead purification and analyzed by using 2μl of DNA template by qPCR. Samples containing more than 4fg of Lxx DNA were classified as Lxx-infected. These cut-off criteria were applied to all samples yielding dichotomous results for both diagnostic assays i.e. “Infected” or “Healthy”. The Receiver Operating Characteristic (ROC) curve is an effective method of evaluating the performance of diagnostic tests enabling the calculation of their specificity (false positive rate), sensitivity (true positive rate), and diagnostic predictive power (Mallett et al., 2012; Trevethan, 2017). The area under the curve (AUC) of a ROC is a measure of a diagnostic test’s predictive power, with higher AUC values indicating the discriminatory accuracy of a test. The RSD-LAMP and qPCR diagnostics tests yielded AUC of 1, thereby suggesting that both diagnostic tests have excellent discriminating power. When assessed independently using the ROC curve, the LAMP reaction time cut-off (LAMP Ct) of 55 minutes was found to be a good discriminator between infected and healthy crops, with a sensitivity of 94.67% and specificity of 100% (**Figures 7A, B**). Similarly, when using a threshold load >4fg of purified Lxx DNA from juice samples in the qPCR assay, the sensitivity and specificity were 98.23% and 100%. However, comparison of the pairwise sensitivity and specificity values across all Lxx DNA concentrations revealed that increasing the threshold above 4fg rapidly reduced the sensitivity of the qPCR assay. In contrast, if a Lxx DNA concentration cut-off of 2fg was used, the sensitivity improved to 100%, with specificity decreasing to 89%. As the aim of the assay is to detect all infected samples, a cut-off of 2fg of Lxx DNA load was chosen for any further analyses.

**Figure 8:**
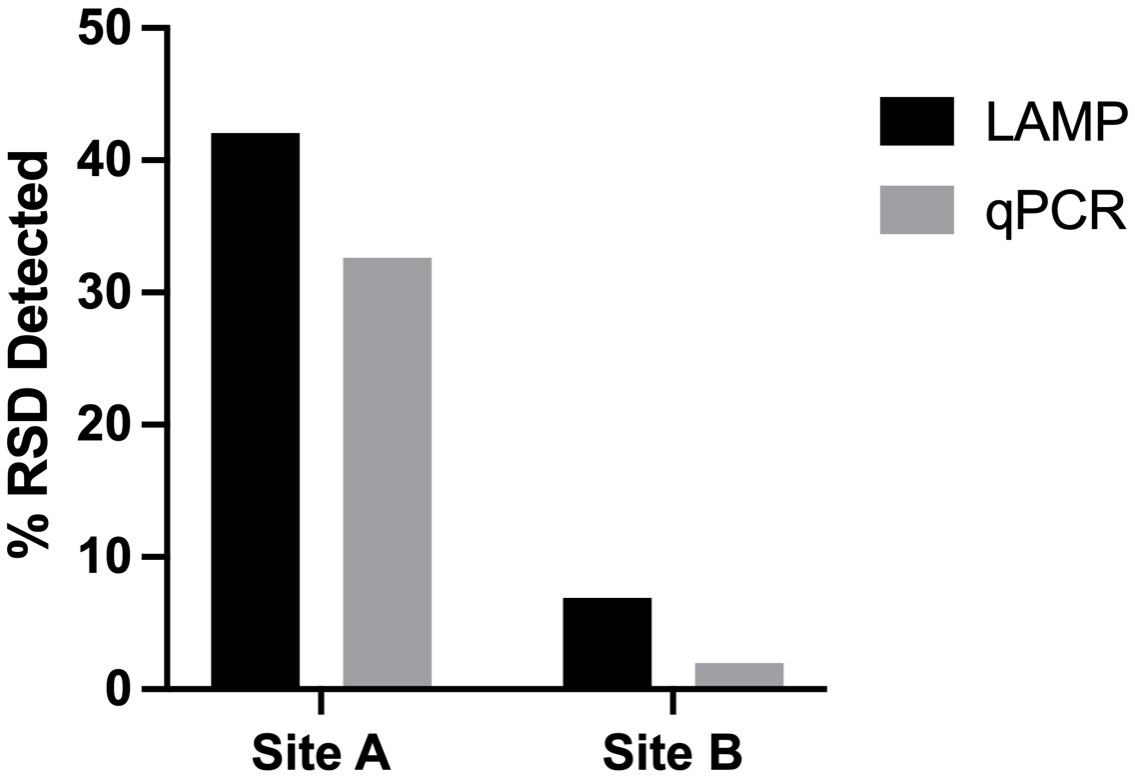
Incidence of RSD across 2 geographically distinct sugarcane milling sites (undisclosed, Australia) detected by the LAMP and qPCR diagnostic methods. Using the newly developed RSD-LAMP assay RSD incidence was calculated at 42.08% at Site A and 6.93% at Site B qPCR-based RSD detection estimated an incidence of 32.62% at Site A and 1.98% at site B.

When the 2 diagnostic methods were compared to each other the newly developed RSD-LAMP assay showed a 77.05% correlation of with the Lxx qPCR assay. The RSD-LAMP assay indicated disease incidences of 42.08% and 6.93% in sites A and B respectively; while the qPCR-based detection method indicated 32.62% and 1.98% disease incidence at Site A and B, respectively (**Figure 8**). Although the differences between the LAMP and the qPCR methods are not significant, the values provided by the -LAMP assay are closer to the known disease incidence in the two growing regions compared to the qPCR assay.

## 4 Discussion

Given the ambiguity of ratoon stunting disease symptoms, its lack of visual diagnostic markers and significant annual sugarcane yield losses caused by the disease, accurate and rapid diagnosis of the causal organism Lxx is essential for the deployment of effective RSD management strategies. Current methods for RSD monitoring are complicated by the sampling procedures. Taking into account that high sugarcane stalk density is 81,000 stalks/ha and that the disease may have limited distribution within a field, it is necessary to carefully design the sampling method and assay a large number of plants to increase the level of confidence for the detection of the disease (Garside and Bell, 2009). Although methods for POC diagnosis of RSD are available (Liu et al., 2013; Ghai et al., 2014), a sensitive assay alone does not facilitate adequate assessment of disease incidence in commercial crops. The problem is compounded by the need to transport samples to specialized laboratories where a large number of assays need to be performed. Our approach seeks to circumvent the above-mentioned problems by detecting Lxx in the crude juice extracted from each crop at the sugar factory, where composite representative juice samples reflect the presence or absence of Lxx in whole crops. A critical advantage of this approach is that a limited number of assays can determine the presence of the disease in a relatively large farm, thus eliminating the need to collect multiple samples and transport them to a laboratory to perform the individual tests. In addition, there are important savings including personnel salary for collection of samples, travel costs to individual farms, transport costs for the samples and the cost of performing thousands of assays at the laboratory. Performing the diagnostic in the crude juice sample will also provide a more accurate indication of the severity of the disease in a field compared to the assay of individual plants, enabling farm and district mapping of the disease in all crops.

**Figure 9:**
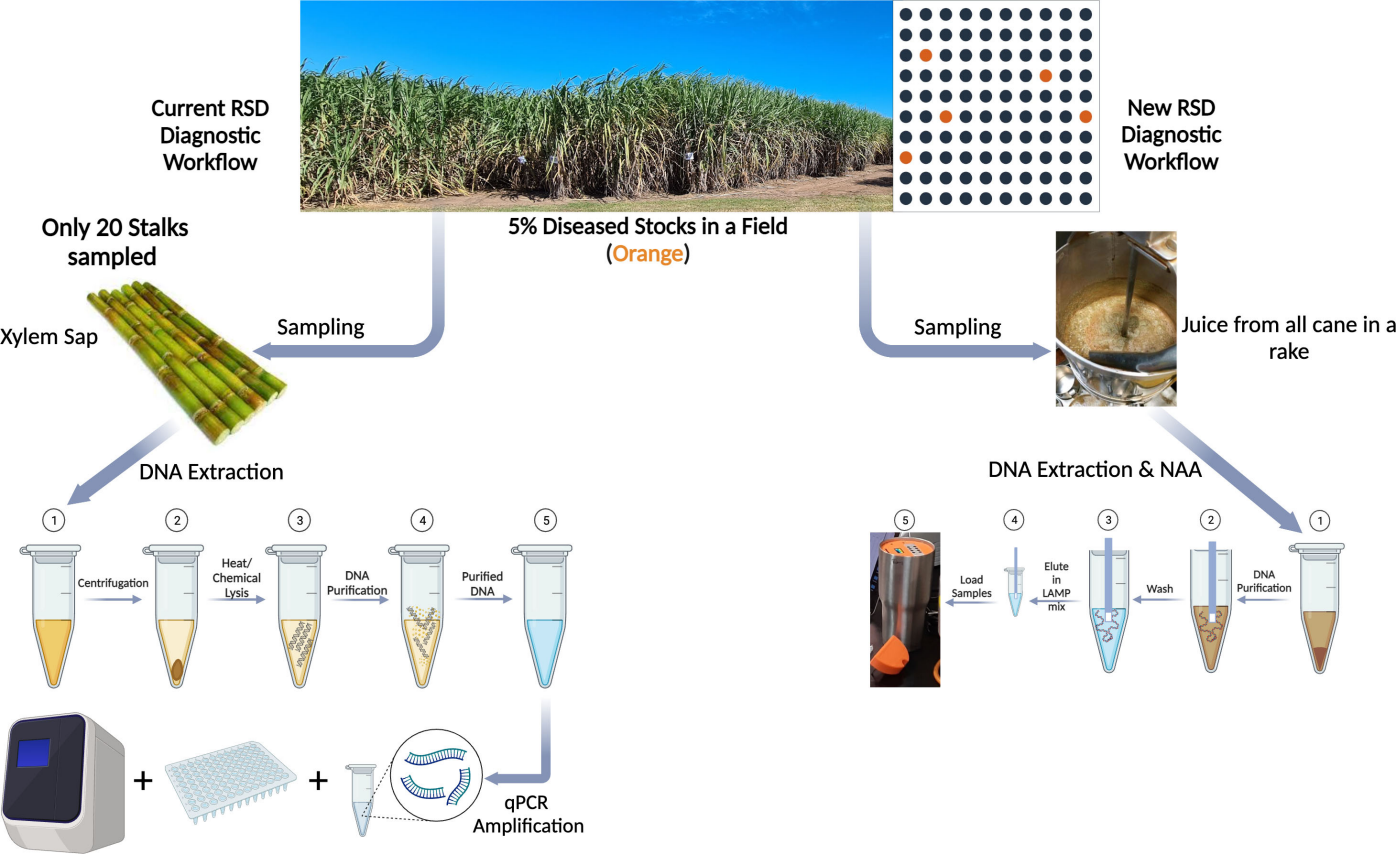
Schematic describing the (left) existing diagnostic workflow for Lxx detection from a sugarcane field v/s the (right) newly developed LAMP based diagnostic workflow (created with BioRender.com). As can be seen, existing workflow involves numerous steps in sample collection, processing and DNA extraction before qPCR based nucleic acid amplification of Lxx, whereas proposed workflow significantly reduces the steps involved in sampling and Lxx NAA. Additionally, in the instance of only 5% Lxx infection in a field, sampling only 20 stalks as part of current workflow could result in inadequate mapping of RSD incidence.

In this study we developed a simplified POC method for reliable and specific detection of Lxx, the causal agent of RSD in sugarcane crude juice which can be performed at the mill. We integrated three simple methods to simplify three bottle-neck issues involved in the development of any POC assay, ie (i) Tissue Sampling, (ii) Nucleic Acid amplification and, (iii) Visualization of assay end-products (**Figure 9**). This was accomplished by using our previously developed nucleic acid dipstick in freshly milled cane juice at mills for DNA recovery, in conjunction with LAMP for amplification, and our custom-made POC device to capture turbidity increases caused by accumulation of magnesium pyrophosphate in the LAMP reaction solution (Zou et al., 2017; Botella, 2022).

LAMP has been widely reported in literature as a suitable method for NAA-based POC detection of human, animal and plant pathogens, with improved or comparable diagnostic sensitivity and specificity to qPCR and nested PCR (nPCR) (Khan et al., 2018; Foo et al., 2020; Inaba et al., 2021; Amoia et al., 2023). In terms of detection limits we found that our LAMP assay was more sensitive than the qPCR assay presently used by the Australian sugar industry. The lower sensitivity of the qPCR assay could be due to loss of target Lxx DNA during the nucleic acid extraction and purification, the presence DNA polymerization inhibitors, or both (Sidstedt et al., 2020). Our findings agree with other reports describing higher sensitivity of LAMP *vs* qPCR (Wu et al., 2018).

As qPCR remains the diagnostic “gold standard”, we validated our LAMP assay using qPCR obtaining a correlation of 77.05%, with the LAMP assay being able to detect Lxx in a larger proportion of crude juice samples. Even though our LAMP assay performed well in non-specialized laboratories at sugar mills during the field-trials, the extreme sensitivity of the assay also presents a challenge to avoid carry-over contamination from samples, which could result in the generation of false positives. This issue can be addressed by either automating the entire workflow with the aid of robotics, or by incorporating CRISPR reagents in the LAMP reaction (Bao et al., 2020).

The NAA based detection methods currently used by the sugarcane industry rely on complicated and time-consuming qPCR based diagnostic methods only suitable for diagnostic laboratory environments; however it provides absolute quantification of pathogen load and is an indirect measure of the extent of infection within a farm (Grisham et al., 2007; Young et al., 2016; Johnson, 2019a; Johnson, 2019b; Andreato et al., 2022). In contrast, our RSD-LAMP assay is a semi-quantitative method, although we observed a positive correlation with the amount of Lxx DNA in crude juice samples, with early signal detection suggestive of high Lxx titer within a sample. The Lxx mill-based LAMP assay using mill juice appears therefore to be a useful tool for assessing crop disease status and it brings crop diagnosis into a practical realm.

The successful implementation of the LAMP assay in mills could allow RSD mapping across districts and regions; the linking of planting and harvesting contractor information to disease incidence may lead to assessing the impact of other farming system and environmental factors on RSD incidence. Issues such as stage in the crop cycle (plant versus ratoon), variety, farming system and RSD integrated-management issues could also be assessed as factors influencing disease incidence.

Future applications of our LAMP based diagnostic platform could include detection of other sugarcane pathogens and pests. This could be facilitated by developing multiplex LAMP and incorporating fluorescent probes like those used in digital droplet PCR for absolute quantification of pathogen loads in a sample. Furthermore, our diagnostic technology can be potentially applied across any crop that is milled for specific pathogen detection like sugar beets, corn, grapes in wine production, etc. Due to the rapid and simple nature of our technology, it can also be used for re-enforcing biosecurity measures of incoming crops or planting materials across the agricultural industry and mitigate any biosecurity incursions.

## Data availability statement

The original contributions presented in the study are included in the article/**Supplementary Material**. Further inquiries can be directed to the corresponding authors.

## Author contributions

SB: Conceptualization, Data curation, Formal Analysis, Investigation, Methodology, Project administration, Validation, Visualization, Writing – original draft. MM: Conceptualization, Data curation, Formal Analysis, Investigation, Methodology, Project administration, Software, Validation, Visualization, Writing – review & editing. JH: Methodology, Writing – review & editing. YZ: Methodology, Writing – review & editing. LG: Methodology, Resources, Writing – review & editing. LM: Resources, Writing – review & editing. RM: Conceptualization, Resources, Supervision, Writing – review & editing. JB: Conceptualization, Funding acquisition, Resources, Supervision, Writing – original draft, Writing – review & editing.
