# Supplementary material for: Changing the diagnostic paradigm for sugarcane: development of a mill-based diagnostic for ratoon stunting disease in crude cane juice
Source: Front Plant Sci. 2023 Oct 12;14:1257894. doi: 10.3389/fpls.2023.1257894 (PMC10613498; doi:10.3389/fpls.2023.1257894)
Supplement: Supplementary file 1 [file DataSheet_1.docx]

Supplementary Figure 1: UDP-N-acetylglucosamine 2-epimerase gene amplified using S1000-2 F3/B3 primer pair, producing an amplicon of 230bp from 13 sugarcane xylem sap samples obtained from 13 different Australian farming sites. With the exclusion of samples 2 and 11, all remaining samples produced a 231bp amplicon. Lane labelled Ladder represents the 1Kb Ladder. Subsequent sequencing of these PCR amplified products divulged comparable homology in the nucleotide sequences of the 11 samples/isolates, thereby suggestive of primer specificity towards more than one *L. xyli* isolate.

Supplementary Figure 2: UDP-N-acetylglucosamine 2-epimerase gene amplified using S1000-2 F3/B3 primer pair, producing an amplicon of 230bp from total DNA extracted from a crude sugarcane juice sample (left; left lane denotes 100bp Ladder). Chromatogram (middle) depicting the DNA sequence of the gene of interest with clean singular peaks, generated by Sanger Sequencing and visualised using Snap Gene software; This DNA sequence was used as a query for a discontiguous blast in NCBI against all available genomes, the highest percentage of sequence identity was shown *by Leifsonia xyli* subsp. xyli str. CTCB07, complete genome (alignment score: 343, % identity: 100%). *Mycrobacterium foliorum* and [*Ruania zhangjianzhongii* strain HY168 chromosome](https://blast.ncbi.nlm.nih.gov/Blast.cgi#alnHdr_1724791821) showed low alignment scores of 56.3 and 53.6, respectively, indicating low sequence homology between the S1000-2F3/B3 amplified DNA and other divergent environmental bacteria.

Supplementary Figure 3: Effect of 0.5M betaine on the specificity of the LAMP reaction in the absence of Lxx DNA. Non-specific amplification with primer set S1000-2 was observed in the LAMP reaction when a final concentration of 0.5M betaine was used. The lane marked “+” denotes positive control with Lxx DNA, and lanes marked “-” denotes no template control i.e. LAMP reaction without target DNA. At 0.5M betaine concentration, non-specific LAMP amplification was observed in 5/7 no template control samples.

Supplementary Figure 4: Effect of primer concentration on the turbidity of LAMP reaction containing purified Lxx DNA. Effect of standard primer concentration (1X) as well as a 2-fold dilution i.e. 0.5X and 0.25X was investigated and the change in turbidity was measured using the portable handheld diagnostic device (n=3).

Supplementary Table 1: Details of the sugarcane xylem samples sourced from *L. xyli* subsp. xyli infected plants from 13 different geographical locations across Australia. The specificity of the S1000-2 primers was determined using these samples. These samples served as a surrogate for the different *L. xyli* subsp. xyli strains.

| Sample # | Location of Sample Collection | Amplicon Produced |
| --- | --- | --- |
| 1 | Rocky Point | Yes |
| 2 | Herbert | No |
| 3 | Mackay | Yes |
| 4 | Maryborough | Yes |
| 5 | Isis | Yes |
| 6 | Burdekin | Yes |
| 7 | Condong | Yes |
| 8 | Tully | Yes |
| 9 | Mossman | Yes |
| 10 | Proserpine | Yes |
| 11 | Bundaberg | No |
| 12 | Innisfail | Yes |
| 13 | Mulgrave | Yes |
