# Supplementary figures and images for: Changing the diagnostic paradigm for sugarcane: development of a mill-based diagnostic for ratoon stunting disease in crude cane juice
Source: Front Plant Sci. 2023 Oct 12;14:1257894. doi: 10.3389/fpls.2023.1257894 (PMC10613498; doi:10.3389/fpls.2023.1257894)

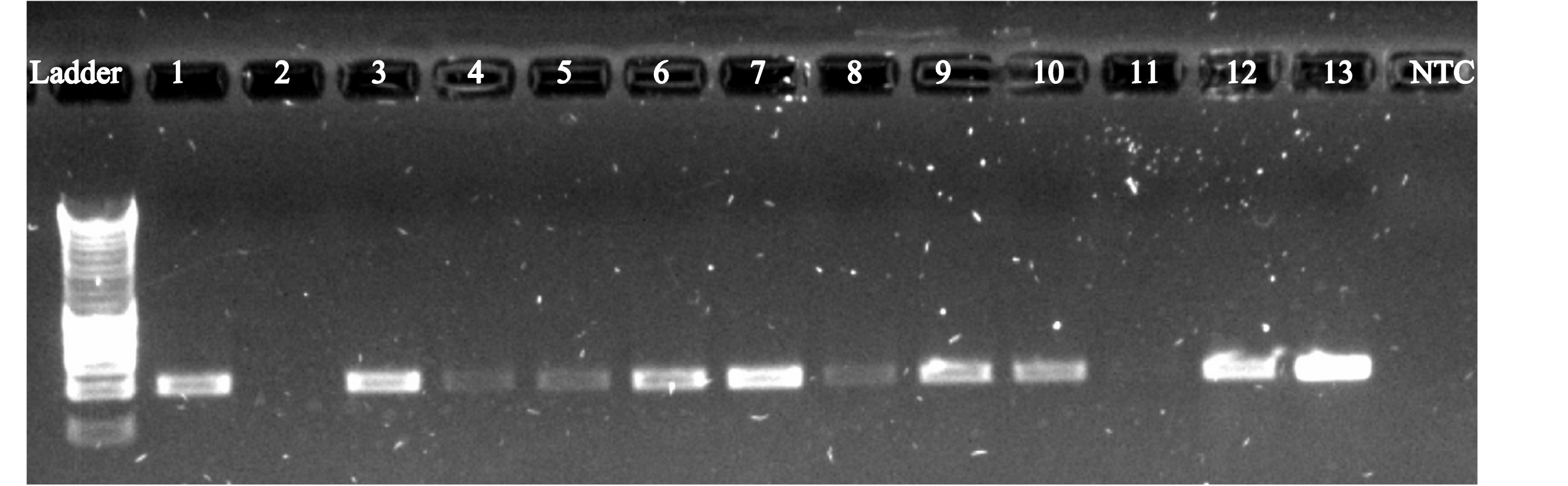

Supplement: Supplementary file 2 [file Image_1.jpeg]

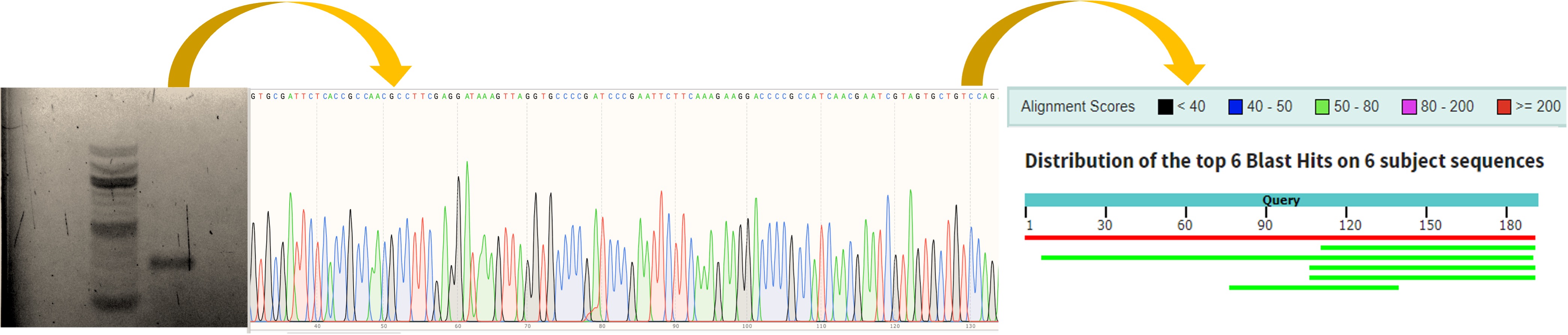

Supplement: Supplementary file 3 [file Image_2.jpeg]

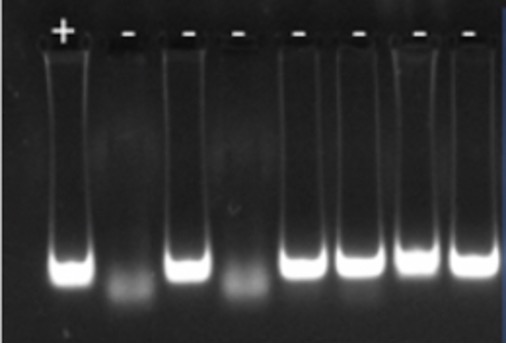

Supplement: Supplementary file 4 [file Image_3.jpeg]

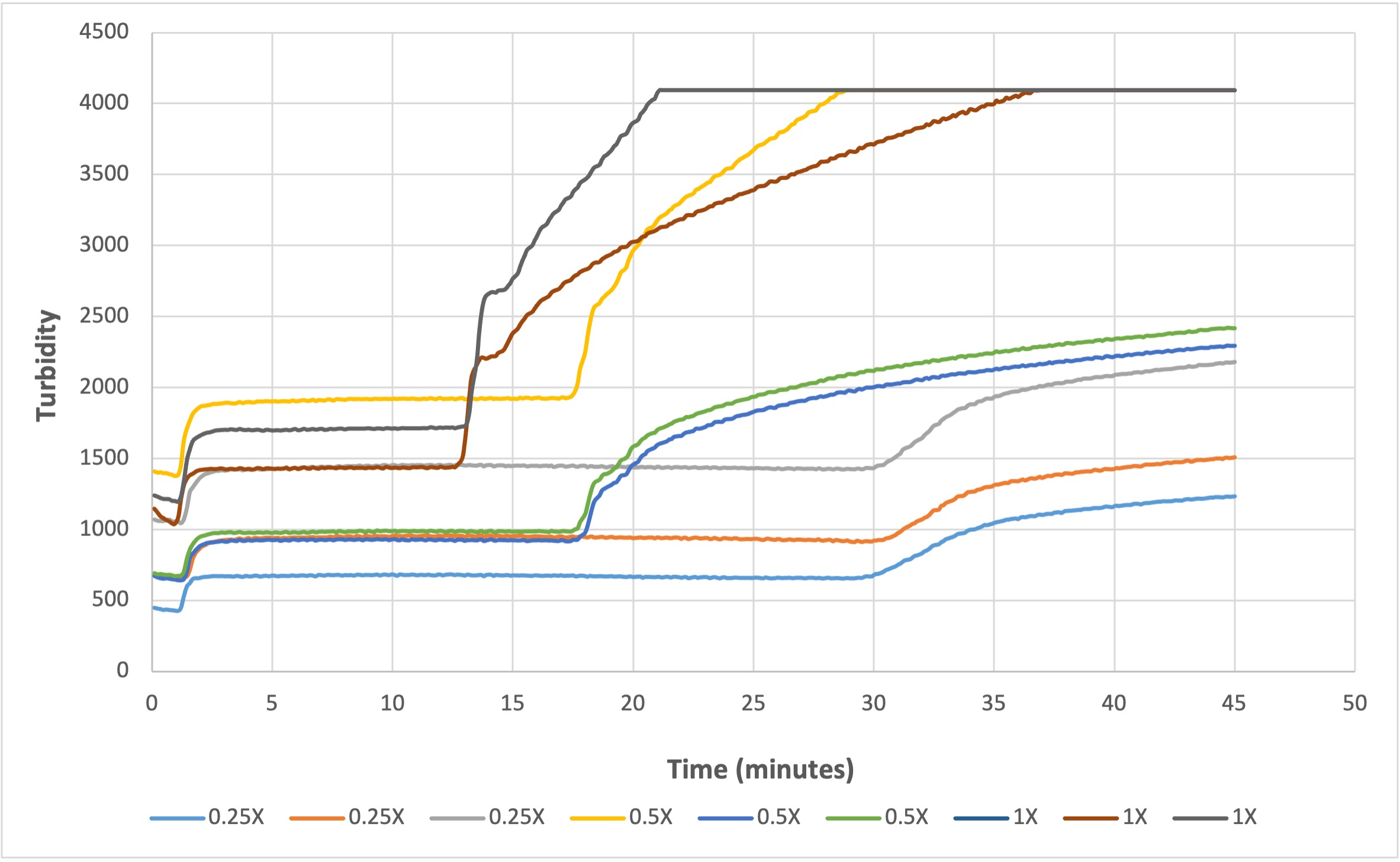

Supplement: Supplementary file 5 [file Image_4.jpeg]
